# Intelligentization helps the green and energy-saving transformation of power industry-evidence from substation engineering in China

**DOI:** 10.1038/s41598-024-59271-5

**Published:** 2024-04-15

**Authors:** Minxin Liang, Lingzi Liu, Weigao Liang, Wei Mi, Kaihui Ye, Jie Gao

**Affiliations:** 1 Guangdong Power Grid Co. Guangzhou Power Supply Bureau, Guangzhou, 510000 People’s Republic of China; 2 China Energy Construction Group Guangdong Electric Power Design & Research Institute Co., Guangzhou, 510000 People’s Republic of China

**Keywords:** Intelligent technologies, Sustainable urban ecology, Substation design, Renewable energy, Energy transformation, Energy and society, Energy economics

## Abstract

The coordinated development of intelligence and greening is an intrinsic demand for high-quality economic and social development. Intelligentization and greening are the leading directions of sustainable development of the power industry. This paper directs of sustainable development of the power industry. This paper empirically analyzes the effect and mechanism of intelligence on the green environmental friendliness of electric power substations by using a panel fixed-effects model and instrumental variable regression, using substation engineering data from China southern power grid during 2013–2022. It is found that the level of intelligence significantly promotes the green performance of substation projects, and this conclusion still holds after a series of robustness tests. Intelligence can reduce material waste and pollutant emissions by improving the engineering environmental monitoring capability and the refinement of engineering resource control, thus improving the environmental friendliness of the project. The research in this paper helps to promote the integrated development of intelligent and green power engineering, to better achieve economic and green goals.

## Introduction

In response to the escalating global trends of climate change and warming, the United Nations convened the Paris Climate Change Conference in 2015. During this pivotal event, representatives from 197 countries achieved consensus on the Paris Agreement, proposing a unified climate response program. In the following years, many countries intensified efforts to reduce carbon emissions by implementing measures such as “certification emission reduction” and “carbon tax” to restrict carbon emissions across various industries and sectors. Among these industries, the power sector, long recognized for its high levels of fossil energy combustion and carbon emissions, must play a pivotal role in emission reduction efforts. It must commit to low-carbon practices and cleanliness to combat climate change, enhance environmental quality, and foster sustainable energy development. Current research predominantly focuses on decarbonization in power generation, with extensive studies conducted on low-carbon technologies for coal-fired power and renewable energy generation. Attention to decarbonization in the transmission and transformation processes, particularly in the construction of power infrastructure, remains limited. As a intersection between the power and construction industries, the construction phase of power projects is integral to the power sector. It entails significant manpower, equipment, material usage, resource consumption, and waste generation, contributing substantially to greenhouse gas emissions. The low-carbon development of substations holds profound implications for energy conservation and emission reduction in the entire power industry. Despite the significance, current low-carbon research mainly concentrates on civil construction, neglecting the imperative of extending such efforts to low-carbon industrial construction. Hence, there is a critical need to explore strategies to facilitate the green and low-carbon transformation of power projects. In this regard, current research has already focused on the construction and implementation of “green” and “zero-carbon” substations. However, overall investment in this area is insufficient. Another issue is that the monitoring and reduction of carbon emissions from these power facilities require further enhancement of the intelligence level of substations.

Now digitization and intelligence continuously drive the transformation of the power industry, fostering the construction of the digital grid and the new power system, thereby facilitating innovation-driven development. In April 2021,China Southern Power Grid issued the “Digital Power Grid White Paper”, advocating for the digital grid as the optimal framework for accommodating the new type of power system. The imperative lies in hastening the construction of the digital power grid, enhancing its operational capabilities, and facilitating the operation, management, and control of a new power system predominantly reliant on new energy sources. Moreover, the introduction of digital demand response alone is projected to decrease the abandonment rate of photovoltaic and wind power in the EU from 7 to 1.6% by 2040, consequently reducing carbon emissions by 30,000 million tonnes, according to predictions by the International Energy Agency (IEA). Additionally, another international consultancy forecasts that global power plant digitization will reach approximately 19% by 2025, enabling power producers to slash operating costs by approximately 27% and thereby reducing carbon emissions from the global power generation sector by 4.7%. While existing evidence underscores the significant support provided by digitization for carbon reduction in power generation, there remains a dearth of attention on whether a similar impact is observed in the transmission and transformation chain. Hence, the central question of interest in this paper emerges: Can intelligence serve as the impetus for the green transformation of power projects? Although many perspectives suggest that enhancing the intelligence level of power projects’ construction is crucial for achieving low-carbon objectives^1^, further theoretical and practical research on this aspect remains limited. Particularly, as the smart grid becomes increasingly intelligent due to the integration of renewable energy sources, other sectors face challenges that necessitate the use of more intelligent equipment and methods to ensure safety. There is still a lack of evidence regarding whether the introduction of intelligent methods can further promote decarbonization of power facilities.

Current research widely acknowledges that the development of intelligent technology will significantly propel the green transformation of the entire economy. Intelligent technology drives enterprises from an “industrial management mode” towards a “digital management mode”^2,3^. This shift encourages companies to actively pursue intelligent production and operation, precise marketing management, and efficient resource management, thereby transitioning product development from experience-driven to data-driven, leading to digital information exchange^4^, and leveraging advantages in reducing R&D costs^5^ to drive green technological innovation within enterprises. Additionally, intelligent transformation can break through resource spatial–temporal boundaries, accelerate knowledge sharing processes, and facilitate the integration of advantageous resources^6^. As digital transformation deepens, enterprises’ organizational management modes, production management modes, and business models undergo disruptive changes, further propelling the transformation and upgrading of traditional industries, changing production methods, and restructuring the entire industry landscape^7^. It is evident that current research mainly focuses on the manufacturing industry, leaving notable gaps in industries with certain public attributes, such as the electricity sector. Numerous studies have delved into intelligent technology, largely based on discussions of national policies and the situation of intelligent technology revolutions in the energy and environmental sectors. Most studies and commentaries concentrate on fault detection and diagnostic technologies^8^, energy planning and prediction models^9^, solar and wind energy forecasting^10^, building energy control^11^, power system optimization^12^, among others.

Within the power engineering field, intelligence methods such as Artificial Neural Networks (ANNs) and fuzzy logic models have been widely applied to address numerous technical challenges in the energy sector^13,14^, including energy market price forecasting^15^, demand-side energy planning^16,17^, energy forecasting^18^, building load management^19^, data security in smart grids and block-chain20, optimization of hybrid systems, renewable energy^21^, and big data management^22,23^, as well as grid fault detection^24^. Research on the transmission and transformation stage, especially in the construction of power projects, is relatively lacking, despite power facilities such as substations contributing significantly to power emissions. Hence, further investigation into the development of intelligent technology in critical areas of power project development is necessary to fully understand its decarbonization potential throughout the power project construction process. By utilizing real-time monitoring, data collection, and intelligent resource scheduling^25^, intelligent technology is poised to enhance the environmental performance of power projects^26^. Stakeholders can identify opportunities to improve efficiency and reduce carbon footprints by analyzing data on energy consumption, material usage, and carbon emissions^27^. In addition to potential environmental benefits, intelligent technology also brings economic advantages to power projects by streamlining processes, optimizing resource utilization^28^, reducing waste, lowering operating costs, and enhancing project efficiency. Despite the significant potential of intelligent technology in power projects, empirical evidence in this area is currently lacking.

Therefore, from the perspective of power projects, this paper will investigate whether intelligent development corresponds to the greening development of power projects and explore its potential mechanisms. This study empirically analyzes the impact of intelligence on the environmental friendliness of substations using a panel fixed-effects model and instrumental variables regression. The analysis is based on existing evaluation standards for intelligence and environmental friendliness of substation projects, utilizing data from 2013 to 2022 from a company within the China Southern Power Grid .The findings suggest that intelligence significantly enhances the greening performance of substation projects, with this conclusion remaining robust. Specifically, intelligence improves environmental monitoring capabilities and enhances the precision of project resource control, thereby promoting the environmental friendliness of the project. Compared with existing research, the marginal contribution of this paper lies in two main aspects. Firstly, it empirically tests the logical fact that intelligence aids in greening development within the field of electric power projects. While existing research has analyzed the green attributes of intelligent development from macro and micro enterprise levels, this paper further focuses on power projects’ analysis and identifies the influence of intelligence on their greening development. Secondly, it reveals the role mechanism of intelligence in facilitating the greening development of electric power projects. Differing from current research, which concentrates on the role mechanism of enterprise intelligence, this paper shifts the focus to power projects and proposes that intelligence enhances environmental monitoring capabilities and resource control precision, thereby promoting their green development. The conclusions drawn from this paper contribute to the expansion of micro-mechanism cognition within the intelligent power industry and aid in facilitating low-carbon power projects. This, in turn, fosters further optimization in carbon reduction deployment within the power industry and facilitates the integration of power project intelligence and greening development.

## Theoretical analysis

### Literature review on the green power project

On the foundation of “green buildings”, studies have explored the requirements and strategic directions for the green and low-carbon development of power projects. This includes concepts such as “green substation” and “zero-carbon substation”. Green buildings are assets that can reduce negative impacts on the natural environment throughout their life cycle stages, including design, construction, operation, maintenance, and demolition^29^. They are characterized by resource and energy efficiency, a preference for renewable energy sources such as wind, solar, and hydroelectric power, pollution and waste reduction, the use of safe and recyclable materials, high environmental quality, residential comfort, and complementarity with the local natural environment and ecology^30^. Green power projects refer to power conversion projects that adhere to energy-saving, environmental protection, and efficient design principles at all stages of planning, design, construction, and operation. They aim to achieve resource conservation, efficiency maximization, sustainable environmental development, and coordinated social progress. Due to the significant functions that green power projects undertake in the power system, they emphasize the integration of safety, quality, environmental protection, and ecology throughout the entire lifecycle of power grid engineering. Driven by technological and managerial innovations, they actively embrace digitization and informatization to accelerate carbon emission reduction. Therefore, green power projects not only need to meet the construction requirements of “green building” but also the construction standards of “green substation”.

Current research emphasizes promoting the green development of power projects through greening measures during the design and construction processes. In terms of design, particular attention should be paid to water supply and drainage systems^31^, optimizing drainage efficiency and enhancing automatic control and sewage recycling to effectively achieve water conservation requirements. Assembled buildings are widely used in substation construction, offering significant advantages in improving construction efficiency and resource conservation, which can reduce operation and maintenance costs in the later stages^32^. Furthermore, the concept of “green lighting” has gained increasing attention in green power projects, enhancing the lifespan of LED light sources and maximizing solar energy utilization to achieve energy-saving and emission reduction goals. Integrating solar photovoltaic power generation into protection chambers also offers significant economic and environmental benefits^33^.

Given the considerable environmental and social benefits of green buildings and green power projects, there have been significant improvements in their development and promotion in various countries, along with relatively mature assessment standards. In 2013, China Southern Power Grid released the first green substation construction standard document in the power industry, namely the “3C Green Grid Construction Evaluation Standard”. Based on green building evaluation standards, this standard provides guidance and evaluation criteria for green substation construction, emphasizing internationalization and the “Four savings and one protection” principle. It covers four key aspects: green planning, green design, green construction, and green delivery^34^. Currently, the overall construction requirements for green power projects are the “Four savings and one protection” principle, namely, saving water, land, materials, and energy during construction and protecting the environment. This requirement has only correspondingly established construction specifications for green construction, while green planning, green design, and green delivery have been neglected. Some scholars have noticed the scope covered by green power projects, establishing evaluation systems spanning multiple stages such as design, construction, and delivery^35^. Some scholars have attempted to use quantitative analysis methods such as Analytic Hierarchy Process^36^ and Fuzzy Objective Element Analysis^37^ to construct indicator systems focusing on the “four savings and one protection” principle. Additionally, some scholars have analyzed the factors affecting the green construction of substations in practice, selecting evaluation indicators from aspects such as planning and management, resource utilization, environmental protection, new technology applications, and ecological benefits, and incorporating digitalization and intelligence indicators^38^.

### Literature review on intelligent enabled green development

Present studies have thoroughly examined the effectiveness and mechanisms of digital technology in promoting economic greening transformation. These analyses combining theory and practice, have identified opportunities and challenges of digital technology from theoretical perspectives. Empirical studies have been conducted to explore the role of digitalization in mechanisms such as green total factor productivity, industrial green transformation, and green technological innovation. From a macroscopic viewpoint, smart technology’s cross-border integration characteristics dismantle previous sectoral boundaries, fostering resource element integration and supply structure optimization. Moreover, it disrupts traditional industrial aggregation patterns, transitioning towards a virtual aggregation mode facilitated by digital technology. This expansion enhances knowledge spillover effects and reduces transaction costs within the economic system. Furthermore, the innovative features of intelligent technology, including human–machine collaboration and group intelligence openness, permeate all economic sectors. These technologies synergize with advanced manufacturing and information and communication technologies to form a ternary interconnected intelligent system termed as “human–machine-object”^39^. This system provides new technical support for green growth, offering fresh impetus for economic transformation.

At the industrial level, existing studies have explored the proposition that big data, as a pivotal component of smart manufacturing, can foster green development through technological innovation, the transformation of traditional industries^40^, and enhancing green total factor productivity. The theory of discontinuous innovation posits that advancements in AI technology lead to discontinuous technological innovations, propelling traditional industries towards intelligent development and giving rise to new formats and industries^41^, thereby fostering green performance. Simultaneously, green technological innovation expedites the substitution of traditional energy-intensive technologies, consequently reducing resource consumption and pollution emissions in production. Moreover, it diminishes information asymmetry, ultimately bolstering green total factor productivity and enhancing the efficiency of resource allocation^42^.

At the micro-enterprise level, intelligence exerts a significant spillover radiation effect on the green innovation behavior of related enterprises, thereby enhancing green technology innovation^43^. For enterprises, replacing traditional, inefficient, and highly polluting technologies through green technology innovation is pivotal for capturing future market opportunities and achieving green development^44^. However, embarking on green transformation entails bearing substantial uncertain risks, with the sizable initial investment posing a formidable challenge for enterprises. This obstacle impedes their departure from traditional paths and hampers their motivation to engage in green innovation activities. Digital technology plays a crucial role in reducing information asymmetry among firms, alleviating financing constraints, and facilitating innovative activities. Additionally, efficient environmental control emerges as a vital avenue for enhancing enterprise green innovation performance. Artificial intelligence technology not only enhances the efficiency of enterprise pollution control^45^ but also enables government environmental protection departments to accurately monitor enterprise pollution emissions. This, in turn, facilitates the formulation of scientific and efficient control measures, thereby fostering positive feedback on enterprise green innovation efficiency. Moreover, digital transformation fosters a conducive innovation ecosystem for enterprises, encouraging active research and development of green technologies and establishing a positive feedback mechanism^46^.

In summary, existing studies have conducted in-depth research on intelligence-enabled greening at both macro and micro levels, and there is a notable gap in research at the project level. Green projects represent on the culmination of specific micro outcomes and the integration of systematic results. Exploring green projects can enrich theories related to intelligent empowerment greening and offer insights into practical green initiatives implementation.

### Theoretical analysis of intelligent assistance for green transformation of electric power project intelligent enhancement of project environment monitoring capability

Traditional project construction objectives typically prioritize hard indicators like cost, progress, and quality, often relegating environmental performance to a soft constraint. However, with the continuous development and application of digital technology, project environmental objectives have become more visible and integrated into all specific aspects of project construction. Consequently, environmental protection requirements now play a more explicit and effective role in project implementation. In this theoretical mechanism, digital technology assumes a crucial role in project construction. It facilitates the establishment of site environmental monitoring systems and the utilization of environmental monitoring electronic signs and alarm systems. These tools enable project management and construction personnel to promptly adjust construction programs to achieve environmental protection objectives and control pollution indicators within specified requirements^47^. Environmental monitoring electronic signs installed at construction sites utilize pollutant detectors to conduct real-time data monitoring and calculations. Various pollution indicators such as air quality, noise levels, and water pollution are measured on-site and displayed at different locations within the construction site. This serves to remind site management and construction personnel to monitor pollution indicators closely and make necessary adjustments to construction activities to keep pollution indicators within the required target range.

Apart from environmental monitoring electronic signage, the environmental alert system represents another important innovation of digital technology in project construction. This system deploys an alarm device, automatically triggering an alarm when pollution indicators at the construction site reach dangerous levels or exceed environmental protection requirements. Consequently, this prompts management and construction staff to take immediate measures to prevent further environmental pollution. This real-time response effectively safeguards the health and safety of the staff and, to some extent, mitigates the impact of environmental pollution on neighboring communities.

Digital technology provides data support and decision-making assistance in the project construction process. Establishing a project environmental data platform and collecting and integrating environmental monitoring data from the construction site enable the formation of a comprehensive environmental performance assessment. These data are instrumental in tracking and analyzing the sources and trends of environmental pollution, serving as an important reference basis for planning and decision-making for similar projects in the future. The data analysis and prediction functions of digital technologies assist managers in predicting environmental changes more accurately and developing more effective countermeasures. Additionally, the application of digital technology promotes information sharing and transparency in project construction. Environmental monitoring data can be transmitted in real-time to regulators and the public, facilitating greater stakeholder participation in the environmental management of the project^48^. Increased public concern and participation in project environmental issues prompt construction units to pay more attention to environmental performance, thereby reducing violations and better achieving sustainable development.

In general, digital technology in construction will be more conducive to realizing environmental objectives. Digital technology means, such as environmental monitoring electronic boards and environmental alarm systems, make environmental objectives visible and integrate them into specific aspects of project construction. They remind and restrain construction management and personnel to adjust construction programs immediately to meet corresponding environmental requirements. Additionally, the data support and decision-making assistance functions of digital technology make project construction more scientific and efficient, laying a solid foundation for sustainable development.

### Intelligent power project resources control refinement

The use of various resources such as construction equipment, materials, and energy in construction projects inevitably results in significant pollutant emissions. This aspect is particularly crucial in substation projects, where resource use and management are vital for reducing emissions and enhancing project greening. Intelligent technology can greatly facilitate project construction, achieving refined resource control and substantial savings in energy, water, and material usage. Intelligent technology optimizes construction planning and resource deployment, leading to more efficient resource utilization^49^. In substation projects, intelligent systems can monitor and predict construction progress to allocate equipment and materials more efficiently, thus avoiding unnecessary resource wastage. Data analysis and algorithm optimization enable project management teams to better anticipate energy, water, and material needs, preventing over-purchasing or overuse. This approach not only conserves resources but also reduces project pollutant emissions. Furthermore, intelligent technologies significantly enhance the energy efficiency of equipment and construction processes. Real-time monitoring by intelligent sensors and systems enables the timely detection and resolution of energy waste issues. Precise control systems adjust equipment operations to maximize efficiency, minimize carbon emissions, and reduce unnecessary energy waste. Moreover, intelligent technology assists project teams in selecting more environmentally friendly and energy-efficient construction materials and equipment, further reducing pollutant emissions. Intelligent lighting systems adjust brightness based on actual needs, while intelligent temperature control systems automatically regulate air-conditioning equipment according to ambient temperatures, minimizing energy waste and unnecessary emissions.

Intelligent technology can also achieve more refined resource management in the construction process. Through IoT technology, real-time monitoring and tracking of construction apparatus and materials can be realized^50^. In this way, the use of resources can be better controlled to avoid waste and over-consumption of resources. Evidence shows that project construction projects applying intelligent technologies have achieved significant improvements in resource utilization and pollutant emissions. By employing data monitoring, optimized management, and refined control, intelligent technologies enhance project sustainability and environmental friendliness. Reducing resource wastage and pollutant emissions not only fulfills environmental protection requirements but also contributes to overall cost reduction.

In summary, the refinement of resource control through intelligent technology can significantly reduce energy, water, and material usage and decrease pollutant emissions in construction management. Measures such as optimizing construction plans and resource deployment, enhancing equipment energy efficiency, and fine-tuning resource management will offer robust support for the greening of project construction. These actions promote project construction toward a more sustainable and environmentally friendly direction of development.

## Methodology

### Modelling

The goal of this paper is to estimate the effect of the level of substation project intelligence on its green performance. The sample comprises all substation engineering projects completed by Company A of the Southern China Power Grid between 2013 and 2022, involving 21 prefecture-level cities in Guangdong Province. Therefore, a two-way fixed effects approach is considered for examination. We set up the estimation model as follows:

1 $$G_{ijt} = a_{0} + a_{1} I_{ijt} + a_{3} X_{ijt} + \theta_{t} + \lambda_{j} + \varepsilon_{ijt}$$

In Eq. (1), $G_{ijt}$ is the greening level of the substation project $i$ built in the year $t$ in the region $j$, $I_{ijt}$ is the intelligence level of the substation project $i$, $X_{ijt}$ is a series of characteristic control variables related to the substation project $i$ , $\theta_{t}$ and $\lambda_{j}$ are the time-fixed effects and region fixed effects, respectively, and $\varepsilon_{ijt}$ is the error term.

Although model (1) carries out controls for two-way fixed effects of time and region, the model may still have endogeneity problems. A substation project with a high level of greening may also have a better level of construction management, and such a project is often more likely to be funded by the government or the power grid company’s smart funding, thus obtaining a higher level of smartness, i.e., there may be an inverse causality problem in the model (1). On the other hand, the environmental performance of project construction is usually a systematic and comprehensive result, and there are many unobservable factors in the process of substation project construction, all of which will affect its green environmental performance, i.e., there may be an omitted variable problem. This article considers using instrumental variable methods to solve the endogeneity problems that may exist in the model (1) and uses a 2SLS method. The model is shown below:

2 $$Phase:I_{ijt} = \beta_{0} + \beta_{1} CI_{ijt} + \beta_{3} X_{ijt} + \chi_{t} + \delta_{j} + \nu_{ijt}$$

3 $$Phase\,II:G_{ijt} = \lambda_{0} + \lambda_{1} \overline{{I_{ijt} }} + \lambda_{3} X_{ijt} + \varphi_{t} + \phi_{j} + \mu_{ijt}$$

In Eq. (2), $CI_{ijt}$ is the instrumental variable for the intelligence level of the substation project $I_{ijt}$, which is measured by the digitization index of prefecture-level cities in this paper (details will be introduced in the subsequent variables section); the estimated intelligent level of the substation project $I_{ijt}$ in Eq. (2) is substituted into Eq. (1) to obtain the estimation model of Eq. (3), in which $\lambda_{{1}}$ is the estimated coefficient of our interest.

### Variables and data

#### Explained variables

The explanatory variable is the level of greening of the substation project. Now the literature ranges from green construction, energy saving and environmental protection, comprehensive benefits, and other aspects of project greening evaluation has launched more research. In terms of practical application, the China Southern Power Grid released a standard that can measure the greening level of substation projects, “3C Green Grid Construction Evaluation Standard” in 2011. It is a systematic evaluation of six dimensions, as shown in Table 1. Each dimension is divided into three grades of low, medium, and high and given different weights, and each grade is set up with several specific evaluation index options (as shown in Table 1). If a project completes the activities required by a specific indicator on a level of a dimension, then it is given a corresponding score, and all the dimension indicators are weighted and summed up according to the weight of the level, that is, the final greening level value of the project is obtained (the same below).

**Table 1:** Evaluation criteria for greening of China Southern Power Grid substation projects.

Evaluation dimension							Rating and weighting	Final score
	Save land d_1_	energy- saving d_2_	water conservation d_3_	save on timber d_4_	eco-friendly d_5_	carry out construction or large-scale repairs d_6_		
N_1_	3	8	2	2	7	3	L_1_ , 1 point each	$G = \sum\limits_{i = 1}^{3} {L*N_{i} }$,where $N_{i} = \sum\limits_{j = 1}^{4} {d_{ij} }$
N_2_	4	10	2	3	9	4	L_2_ , 2 points each	
N_3_	4	12	3	4	11	4	L_3_ , 3 points each	

Sourced from “3C Green Grid Construction Evaluation Standard”, specific activities required by the evaluation indicators can refer to this standard. This paper will not make a specific introduction due to space limitations.

#### Explanatory variables

The explanatory variable is the level of substation project intelligence. The existing studies mainly measure the level of intelligence from two aspects. One is the method of constructing an indicator system represented by Sun & Hou^51^, which constructs the indicator system for measuring the level of industrial intelligence from three aspects: infrastructure, production application, competitiveness, and benefits. The second is the industrial robot input measurement proposed by Song & Zuo^52^. Both of these methods measure the level of regional intelligence, while the research subject is a single substation project in this paper, so we refer to the evaluation method for the intelligence of substation projects in the “3C Green Grid Construction Evaluation Standard”, which is carried out from the four dimensions of intelligent primary equipment, secondary equipment and its network, other secondary systems, and intelligent advanced application. Each dimension is also divided into three levels of low, medium, and high and given different weights set up with several specific evaluation index options (as shown in Table 2). If a project completes the activities required by a specific index on a level of a dimension, then it is given a corresponding score, and all the dimension indexes are weighted and summed up according to the weights of the level to obtain the final value of the project’s intelligence level.

**Table 2:** Evaluation criteria for intelligence of China Southern Power Grid substation projects.

Evaluation dimension					Rating and weighting	Final score
	Intelligent primary equipment f_1_	Secondary equipment and its network f_2_	Other secondary systems f_3_	Intelligent Advanced Applications f_4_		
M_1_	2	4	3	2	L_1_, 1 points each	$G = \sum\limits_{i = 1}^{3} {L*M_{i} }$, where $M_{i} = \sum\limits_{j = 1}^{4} {f_{ij} }$
M_2_	3	5	4	3	L_2_, 2 points each	
M_3_	4	6	4	4	L_3_, 3 points each	

#### Mechanism test variables

Environmental monitoring capability for substation project. The metrics included the number of environmental indicators monitored on-site, the number of monitoring electronic boards displaying locations, the hierarchy of site personnel aware of environmental objectives, the frequency of managers viewing environmental indicators, and the frequency of construction workers viewing environmental indicators. The calculation method is borrowed from Bloom & Van Reenen^53^ and Brynjolfsson & McElheran^54^, etc., by assigning equal spacing of 0–1 points to the response options for each question in descending order (For example, for the question “Number of environmental indicators monitored at the project site”, the answers were assigned as 0, 1/3, 2/3, 1), and finally synthesizing the five questions into a 0–1 point environmental monitoring capability indicator by arithmetic mean. Table 3 shows the specifics of the indicators and the criteria for judging them.

**Table 3:** Measures of environmental monitoring capacity.

serial number	Content of the indicator	Evaluation options
1	The number of environmental indicators monitored at the project site (e.g. PM2.5, NO_2_, noise, etc.)	① None; ② 1–2; ③ 3–5; ④ 5 or more
2	Monitoring of the number of electronic signage roll-out locations	① None; ② 1; ③ 2–3; ④ More than 3
3	Site personnel are aware of environmental objectives	① No target; ② There is a goal but no one knows about it; ③ There are objectives known only to the management; ④ There is a target, but no one knows about it; ④ There is a target, but only the management knows about it
4	Frequency of managers looking at environmental indicators	① No indicator; ② There are indicators but basically no view; ③ Weekly view; ④ Viewed daily; ⑤ Viewed multiple times per day
5	Frequency of construction workers viewing environmental indicators	① No indicator; ② There are indicators but basically no view; ③ Weekly view; ④ Viewed daily; ⑤ Viewed multiple times per day

Refined control of substation project resources. This paper measures the frequency of statistical inventory of construction material usage, the frequency of construction energy consumption monitoring and auditing, and the frequency of construction water consumption monitoring and auditing. The method of calculation is the same: the answer options for each question are assigned an equidistant score of 0–1 in descending order, and finally the five questions are synthesized by arithmetic average into an environmental monitoring capacity indicator of 0–1 points. Table 4 shows the specifics of the indicators and the criteria for judging them.

**Table 4:** Measures of resource control refinement.

Serial number	Content of the indicator	Evaluation options
1	Frequency of statistical inventory of construction material usage	① No statistics; ② Statistics but basically no inventory; ③ Monthly inventory; ④ Weekly inventory; ⑤ Daily inventory
2	Frequency of construction energy consumption monitoring audits	① No monitoring; ② Monitored but largely unchecked; ③ Semi-annual or quarterly audit; ④ Monthly audit; ⑤ Weekly audit; ⑥ Daily audit
3	Frequency of construction water monitoring audits	① No monitoring; ② Monitored but largely unchecked; ③ Semi-annual or quarterly audit; ④ Monthly audit; ⑤ Weekly audit; ⑥ Daily audit

#### Instrumental variables

We choose to utilize the city digitization index of prefecture-level cities compiled by Tencent, one of the largest Internet companies in China, as an instrumental variable for assessing the intelligence level of each substation project. Tencent, being one of China’s largest Internet companies, has been compiling the “Digital China Index” since 2013. This index is based on big data collected from Tencent and its numerous industrial chain partners, measuring the degree of digital development across Chinese cities from various dimensions, including industry, consumption, government affairs, and culture. A reasonable instrumental variable needs to satisfy two conditions: firstly, the explanatory variable must have a strong correlation with the instrumental variable; secondly, the instrumental variable should not be correlated with the explanatory variable, adhering to exogenous assumption requirements. In terms of the relevance condition, regional digitization typically relies on industry digitization, which also provides favorable institutional conditions and infrastructure for the intelligent transformation of regional substation projects. Hence, it can be inferred that regional digitization strongly correlates with the intelligence level of local substation projects. Concerning the exogenous assumption requirement, regional digitization is not directly linked to the environmental performance of substations. Thus, we consider the regional digitization index as meeting the conditions of a reasonable instrumental variable. Additionally, we conduct tests to evaluate the applicability and reasonableness of the instrumental variable in the subsequent section.

#### Other control variables

The intelligent and green transformation of substation projects is influenced by various factors, including the characteristics of the projects themselves and their geographical locations. This study aims to control for these factors through the following measures: (1) Scale of project investment^55^.The enhancement of green performance in engineering projects requires significant capital investment. Implementing green and sustainable practices in projects is often associated with higher costs and risks, impacting economic performance^56^. Only projects with larger investment scales can achieve this and are better positioned to drive green construction. (2) Type of project construction: Different types of projects, such as new construction, renovation projects, expansion projects, and relocation projects, prioritize sustainable development differently and face varying policy requirements and costs^57^. This study controls for project types using fixed effects, assigning values of 1, 2, 3, and 4 to new construction, renovation projects, expansion projects, and relocation projects, respectively. (3) Project construction cycle: Generally, projects with longer durations attract higher investments. They exhibit a stronger inclination and scope to apply digital technology compared to short-term projects, while also focusing on the green development benefits of the project. (4) Geographic location: Green transformation exhibits typical externalities, necessitating the implementation of government environmental regulations and policies. Environmental regulation policies in China are unequally implemented across regions, with the environmental regulations in the central and western regions being relatively less stringent compared to those in the eastern region^58^. Additionally, factors such as the level of economic development, environmental awareness, and natural conditions vary across regions and significantly influence the green performance of projects. This study focuses on all substation projects completed by Company A of the China Southern Power Grid from 2013 to 2022, covering 21 prefecture-level cities in Guangdong Province. Regional fixed effects are considered to control for economic, social, and other factors in the project locations.

The data comes from all substation projects completed by A company of China Southern Power Grid from 2013 to 2022, and Table 5 shows the descriptive statistics of the main variables.

**Table 5:** Descriptive statistics of the main variables.

variant	Sample size	Average value	(Statistics) standard deviation	Minimum value	Maximum values
Explanatory variable					
project Greening	2642	86.536	26.353	25	114
Explanatory variable					
project Intelligence	2642	27.725	14.632	11	54
Mechanism explanatory variables					
Environmental monitoring capabilities	2642	0.627	0.013	0	1
Fine-tuning of resource control	2642	0.562	0.015	0	1
Instrumental variable					
Regional digitalization Index	2642	0.562	0.124	0.152	0.896
Control variable					
Scale of project investment (take pairs)	2642	8.365	2.373	6.573	12.473
project and construction cycle	2642	15.323	5.725	4	36
Type of construction	2642	2.132	0.531	1	4

## Results

### Baseline regression results

#### OLS regression results

Based on the above data, Table 6 shows the results of OLS regression.

**Table 6:** OLS regression results.

OLS	(1)	(2)	(3)
project Intelligence	5.246***	4.725***	4.725***
	(0.836)	(1.163)	(1.163)
The scale of project investment			0.135**
			(0.068)
project and construction cycle			− 0.112
			(0.075)
Type of construction	N	N	Y
area fixed effect	N	Y	Y
time fixed effect	N	Y	Y
R2	0.023	0.725	0.762
N	2642	2642	2642

Robust standard errors in parentheses, *, **, and ***Indicate significance at the 10%, 5%, and 1% levels, respectively (followed the below).

The results in column (1) indicate a significant positive correlation between the level of engineering intelligence and its green level, suggesting that enhancing the application of intelligent technology in engineering can significantly promote the improvement of green performance. Even after controlling for regional and time fixed effects, column (2) still presents a significant positive correlation. In column (3), after introducing relevant control variables, the results remain robust.

#### IV regression results

To address endogeneity, based on the above data, Table 7 shows the results of IV regression.

**Table 7:** IV regression results.

	Phase I	Phase II
IV-OLS	project Intelligence	project Greening
project Intelligence		4.836***
		(1.326)
Regional Digital Index	6.836***	
	(0.846)	
control variable	Y	Y
area fixed effect	Y	Y
time fixed effect	Y	Y
R2	0.738	0.746
N	2642	2642
Kleibergen-Paap rk LM statistic	83.847***	
Cragg-Donald Wald F-statistic	74.649***	

Control variables include project investment scale, project construction period, and project construction type (followed below).

Utilizing Models (2) and (3) for 2SLS regression, the results are presented in Table 7. The estimates from the two-stage regression are largely consistent with the baseline regression results, indicating that the digitalization of management in power grid enterprises promotes the enhancement of their precision investment efficiency. Moreover, the instrumental variables also pass the overidentification test and weak instrumental variable test, suggesting sufficient identification of endogeneity issues within the model.

### Robustness tests

#### Instrumental variables exogenous

While this paper logically analyzes the exogeneity of instrumental variables, statistical validation of the exogeneity assumption cannot be confirmed under the ‘just identification’ scenario. Therefore, further discussion on the exogeneity of instrumental variables is warranted. Firstly, this study regresses the regional digitization index against the level of greenization in substation engineering. Table 8, column (1), suggests that the regional digitization level may significantly influence the greenization level of substation engineering. This paper proceeds to regress both the regional digitization index and the level of engineering intelligence against the greenization level of substation engineering. Results in column (2) of Table 8 show no substantial difference compared to the baseline regression. It can be concluded that the instrumental variables selected in this study still meet the exogeneity assumption requirements.

**Table 8:** Exogeneity test for instrumental variables.

OLS	(1)	(2)
Regional Digital Index	2.532***	0.004
	(0.451)	(0.042)
project Intelligence		4.686***
		(1.158)
control variable	Y	Y
area fixed effect	Y	Y
time fixed effect	Y	Y
R2	0.635	0.643
N	2642	2642

#### Further robustness tests

First item: Delete Guangzhou and Shenzhen, two economically advanced sub-provincial cities. On the one hand, Guangzhou and Shenzhen are highly developed in terms of economic development, and have obvious advantages in technological innovation and intelligent development; on the other hand, the two cities are sub-provincial cities at a level higher than that of other prefecture-level cities and usually enjoy more favorable treatment and support in terms of policies and resources. The development of power projects in these two cities is quite different from other cities.

Second item: Remove the impact of the shock of the New Crown epidemic. Deleting the sample of 2020–2022: Considering that the selected sample interval contains 2020 and 2021, China’s infrastructure construction has been subject to strict external shocks due to the New Crown Pneumonia epidemic, and the investment in the electric power project has also been greatly affected. Therefore, the special samples of these two years are removed and regressed again, as shown in columns (2) to (4) of Table 9.

**Table 9:** Further robustness tests.

	(1)	(2)	(3)
IV-OLS	Exclude samples from sub-provincial level cities	Stripping out the impact of the epidemic	Controlling for the digitalization policies of enterprises
project Intelligence	3.462***	3.865**	4.253***
	(1.153)	(1.182)	(1.164)
control variable	Y	Y	Y
area fixed effect	Y	Y	Y
time fixed effect	Y	Y	Y
R2	0.814	0.826	0.723
N	1896	1636	2642

Control variables include the total annual power generation of the enterprise, staff size of the enterprise, number of patents applied by the enterprise, power supply structure of the grid, regional industrial structure, and regional urbanization level.

Third item: Further control for the impact of corporate digitization strategy. China Southern Power Grid began to focus on the digital transformation of the grid in 2019, promoting continuous innovation in corporate management, services, and business models, and achieving significant results in the areas of smart distribution grids and digital platforms, which are controlled in this paper by adding a time dummy variable for policy shocks to the regression model.

Tables 8 and 9 report the robustness test results which show that intelligence has a significant contribution to the green development of grid projects under the linear model, which verifies that the results are robust.

## Mechanisms

To further understand how intelligence affects the green transformation development of power grid projects, and to provide optimized paths and differentiated implementation solutions for digital technology to better empower the green development of power grid projects, it is necessary to carry out mechanism analysis. From the previous theoretical analysis, it can be seen that intelligent technology acts on green economic growth through two paths environmental monitoring intelligence and resource control refinement in grid projects. For this reason, this paper uses the mediating effect three-step method to identify the mechanism.

### Environmental control mechanisms

The mediating effect regressions for the environmental monitoring mechanism are presented in Table 10, where columns (1) and (3) show the regression results before and after the inclusion of environmental monitoring as a mechanism variable, respectively, and column (2) reports the effect of intelligence on the environmental monitoring capability. The results show that environmental monitoring capability is an intermediate path for intelligence to influence the greening of grid project. In particular, the partial mediating effect of this path is 45.51%, which indicates that the mediating effect of the environmental monitoring mechanism is stronger in the growth effect of the green grid projects, which will increase the greening level of the project by 1.752% at a 1% significance level. On the one hand, the current artificial intelligence in the grid project further expands the application scenes and application scope of intelligent services, realizes real-time supervision and full control of the environment at the construction site, and promotes the precision and automation of the green construction management; on the other hand, a smart platform centered on digital technology can provide the project management with environmental performance data and carbon emission traceability information, and synchronously share the information on environmental pollution with the stakeholders, and externally control the greening of the power grid project from the outside. On the other hand, the intelligent platform developed based on digital technology can also provide environmental performance data and carbon emission traceability information for project management, share environmental pollution information to all stakeholders synchronously, and give environmental performance targets to the grid project construction from the outside, providing multiple guarantees for the green and low-carbon development of grid projects.

**Table 10:** Environmental Monitoring Mechanism Test. Control variables include the total annual power generation of the enterprise, staff size of the enterprise, number of patents applied by the enterprise, power supply structure of the grid, regional industrial structure, and regional urbanization level.

	(1)	(2)	(3)
IV-OLS	project Greening	environmental monitoring	project Greening
Project INTELLIGENCE	4.836***	0.735***	2.635***
	(1.326)	(0.062)	(0.656)
environmental monitoring			1.752***
			(0.086)
control variable	Y	Y	Y
area fixed effect	Y	Y	Y
time fixed effect	Y	Y	Y
R2	0.746	0.782	0.753
N	2642	2642	2642

**Table 11:** Resource control mechanism test.

	(1)	(2)	(3)
IV-OLS	Project greening	Resource management	Project Greening
Project intelligence	4.836***	1.824***	3.251***
	(1.326)	(0.412)	(1.852)
Resource management			2.246***
			(0.624)
Control variable	Y	Y	Y
Area fixed effect	Y	Y	Y
Time fixed effect	Y	Y	Y
R2	0.746	0.724	0.758
N	2642	2642	2642

Control variables include the total annual power generation of the enterprise, staff size of the enterprise, number of patents applied by the enterprise, power supply structure of the grid, regional industrial structure, and regional urbanization level.

### Resource control mechanisms

Similarly, Table 11 reports the regression results of resource control mechanisms, which shows that the influence effect of intelligence in the development of grid project greening is significantly positive, and the regression coefficient of intelligence on project greening decreases from 4.836 to 3.251 after adding the mediating variable of the refinement of resource control, whereas the influence effect of resource control on project greening is significantly positive, with a regression coefficient of 2.246, and the partial mediating effect of the resource control mechanism is 32.78%. It indicates that intelligent technology can indeed promote the green construction of power grid projects by improving the refinement of resource control in the construction of power grid projects. On the other hand, from the partial mediation effect in Table 10 and Table 11, it can be seen that the mediation effect of environmental monitoring ability is stronger than that of resource control, which may because existing intelligent technology applied to real-time monitoring and analysis is more mature, and the practice of the grid project has a better application effect, and due to the constraints on the diversity of resource constraints and demands and the coordination of multiple departments, the practical application of resource control still has certain difficulties. Control still has certain difficulties and risks.

## Conclusions

### Findings and insights

Based on the fact that the power industry accelerates green transformation, this paper further focuses on the green performance of power projects, studies whether the current trend of intelligence can influence the greening development of power projects, and explores the mechanism of its role. The conclusions of this paper are as follows: (1) Intelligence significantly promotes the enhancement of the green development level of power grid projects, and this effect remains valid when considering endogeneity and robustness testing; (2) Intelligence can indirectly enhance the green development level of power grid projects by strengthening the environmental monitoring capability of the project site and improving the degree of refinement of resource control, with the mediating effect of environmental monitoring capability stronger than the degree of refinement of resource control. Based on the aforementioned research findings, this paper proposes policy insights focusing on the five aspects: (1) Accelerating the deep integration of intelligent technology with power engineering. Digital technology exhibits promising application scenarios and roles within power engineering. It need further leverage digital technologies such as big data, 5G, industrial Internet, cloud computing, artificial intelligence, and digital twins to monitor and transform the entire process of engineering construction towards greener practices, thereby maximizing the cost reduction and energy-saving attributes of intelligence. (2) Enhancing financial support and policy guidance for the intelligent development of power engineering, further emphasizing the pivotal role of environmental monitoring and resource control in project construction, gradually replacing traditional means of project construction to achieve large-scale, intensive effects. Currently, there is limited policy impetus for promoting green substations. These substations lack market-driven competition and positive impetus for transformation due to their public attributes. This underscores the necessity of government support in facilitating this transition. (3) Strengthening green project management practices in power engineering. Focusing on energy management, lean planning, and green construction, among other typical scenarios, utilizing intelligent means to enhance the project’s overall fine management, conducting green energy monitoring and evaluation, providing standardized management digital carbon reduction solutions for the green development of the power industry, and offering an operational and practicable path reference for the green transformation of more industrial enterprises. (4) Reinforcing awareness of green construction among enterprises. Currently, management concepts within construction enterprises are outdated, and their comprehensive quality is relatively low. Managers typically employ traditional management models and lack innovative and green construction concepts. Therefore, to ensure the success of green power engineering construction, it is imperative to change the backward management concepts of managers and enhance their comprehensive quality. Considerable technical training should be provided to construction personnel, integrating green construction concepts into the training, enhancing their understanding of green construction, and ensuring actual implementation of green construction practices during construction. (5) Establishing a sound legal and regulatory system for green engineering construction. Individuals and enterprises violating relevant laws and regulations should be subject to corresponding legal penalties to ensure the implementation of the legal system. Additionally, the government should strengthen supervision by professional institutions to evaluate the degree of green management in construction projects. Through scoring indicators, the implementation of green management in these projects can be reflected.

### Limitations and future research directions

The research has certain limitations. Firstly, due to constraints on data availability, the study focused solely on power engineering projects in Guangdong Province, without including projects from other regions. Variations in geographical locations may affect the effectiveness of engineering intelligence and greening differently. Future investigations should explore the influence of external factors, such as the level of intelligent development in different regions. Secondly, while the evaluation method for the green level of power engineering projects used in this study is relatively mature, it lacks a post-evaluation phase. Therefore, it fails to form a complete evaluation loop and lacks comprehensiveness in the evaluation system. Future research should integrate the entire life cycle of projects into the green performance evaluation index, further examining green power engineering from the perspective of operation and maintenance. Another notable limitation pertains to the evaluation of intelligence and greening in power engineering projects. The absence of relevant evaluation standards necessitated reliance on criteria established in 2011, posing a timeliness issue. Lastly, the scarcity of literature on related topics during the writing process indicates a weak foundational basis for this study, highlighting the need for further exploration of underlying mechanisms in future research.

## Data Availability

The data presented in this study are available on request from the corresponding author.
